# Significance of Atrial and Brain Natriuretic Peptide Measurements in Fetuses With Heart Failure

**DOI:** 10.3389/fphys.2021.654356

**Published:** 2021-03-18

**Authors:** Takekazu Miyoshi, Hiroshi Hosoda, Naoto Minamino

**Affiliations:** ^1^Department of Regenerative Medicine and Tissue Engineering, National Cerebral and Cardiovascular Center, Suita, Japan; ^2^Clinical Research Center, National Center for Child Health and Development, Tokyo, Japan; ^3^Omics Research Center, National Cerebral and Cardiovascular Center, Suita, Japan

**Keywords:** natriuretic peptide, fetal heart failure, congenital heart defect, arrhythmia, placenta, metabolism, prenatal diagnosis, ultrasonography

## Abstract

Fetal heart failure is mainly caused by congenital heart defect and arrhythmia. It is difficult to appropriately diagnose the severity of fetal heart failure simply by ultrasonography because the development of a fetal heart in fetoplacental circulation and how well the fetal myocardium can adapt to postnatal cardiopulmonary circulation are challenging to assess. In adult cardiology, natriuretic peptides (NPs) are the most useful biomarker of heart failure; however, studies investigating NP levels in the fetuses and amniotic fluid are quite limited. Furthermore, little is known about their production and metabolism. This review summarized the most relevant findings on NP levels in the umbilical cord blood and amniotic fluid. The findings can then extend their use as a diagnostic biomarker of heart failure in fetuses with congenital heart defect and/or arrhythmia.

## Introduction

Prenatal diagnosis of complex congenital heart defects (CHDs) is critical to predict the need for emergent postnatal interventions and facilitates a more rapid stabilization of postnatal circulation (Levey et al., 2010; Holland et al., 2015). The pathophysiology of CHDs, including Ebstein’s anomaly, is associated with a high perinatal mortality caused by the progression of heart failure *in utero* (Freud et al., 2015). Sustained tachyarrhythmia or bradyarrhythmia is associated with the progression of fetal heart failure (Schmidt et al., 1991; Naheed et al., 1996). Therefore, transplacental antiarrhythmic therapy for fetal tachyarrhythmia is highly recommended during preterm gestation (Donofrio et al., 2014). An accurate prenatal diagnosis of structural heart abnormalities or arrhythmias and an assessment of heart failure *in utero* are important in providing appropriate management for fetuses with CHD and/or arrhythmia (Miyoshi et al., 2019c).

The development of a fetal heart in fetoplacental circulation and how well the fetal myocardium can adapt to postnatal cardiopulmonary circulation are difficult to assess. Thus, fetal heart failure is still challenging to diagnose. Recent studies have shown that the cardiovascular profile score is a comprehensive echocardiographic marker of fetal heart failure (Falkensammer et al., 2001; Huhta, 2005; Hofstaetter et al., 2006). This method is based on a composite scoring system for grading the severity of fetal heart failure using five echocardiographic parameters that are as follows: fetal effusion, arterial and venous Doppler findings, heart size, and cardiac function. The cardiovascular profile score may be useful in the baseline and serial evaluations of fetuses at risk of myocardial dysfunction. However, there are some issues associated with the use of echocardiography for evaluating cardiac function in fetuses with CHD and/or arrhythmia (Wieczorek et al., 2008; Miyoshi et al., 2019c). For example, the severity of tricuspid valve regurgitation is not always easy to evaluate accurately (Neves et al., 2014). The gap in timing between atrial contraction and atrioventricular valve closure due to arrhythmias leads to abnormal venous Doppler findings. Therefore, it is important to identify objective biomarkers that can reflect the severity of fetal heart failure.

Natriuretic peptides (NPs) are established as biomarkers of heart failure in adult cardiology (Böhm et al., 2011; McMurray et al., 2012); however, studies investigating NP levels in the fetuses and amniotic fluid are quite limited. Furthermore, little is known about their production and metabolism. This review summarized the most relevant findings on NPs in the umbilical cord blood and amniotic fluid. This finding can extend their use as a diagnostic biomarker of heart failure in fetuses with CHD and/or arrhythmia.

## Clinical Significance of NPs As Biomarkers of Fetal Heart Failure

### Umbilical Cord Blood NP Levels

Our previous study showed that plasma NP levels in the umbilical cord blood were correlated with the severity of heart failure in fetuses with CHD and/or arrhythmia (Miyoshi et al., 2018b; Table 1). The plasma concentrations of atrial NP (ANP), brain NP (BNP), and N-terminal fragment of pro-brain NP (NT-proBNP) in the umbilical cord blood had similar profiles in heart failure. Several studies showed that fetuses with CHD have significantly higher NT-proBNP levels in the umbilical cord blood than controls (Lechner et al., 2009; Merz et al., 2012; Bae et al., 2015; Lee et al., 2016). The NT-proBNP levels in the umbilical cord blood of fetuses with a single ventricular physiology are significantly higher than those with a biventricular physiology. Fetuses with a ventricular outflow tract obstruction and an intact interventricular septum have significantly higher NT-proBNP levels than those with other types of CHD. Hence, a high ventricular pressure leads to increase NP levels (Merz et al., 2012; Bae et al., 2015). We analyzed in detail the association between heart failure severity and NP concentrations. Results showed that, compared with other types of CHD, right heart defects with moderate or severe tricuspid valve regurgitation, including Ebstein’s anomaly, are associated with lower cardiovascular profile scores and higher NP levels (Miyoshi et al., 2018b). Conversely, fetuses with a right heart defect but no or mild tricuspid valve regurgitation, which does not lead to high right ventricular pressure, had low NP levels. These findings strongly support the notion that elevated NP levels are mainly attributed to a high central venous pressure, rather than morphological abnormality itself.

**Table 1:** ANP, BNP, and NT-proBNP in the umbilical cord blood and amniotic fluid in fetuses with CHD/arrhythmia.

	ANP	BNP	NT-proBNP
**Detection specificity**	Mature ANP proANP	Mature BNP proBNP	NT-proBNP proBNP
**Umbilical cord blood**			
Concentration, median (minimum–maximum)^*^	42 (6–1,975) pg/ml	18 (0.2–1,276) pg/ml	636 (140–24,921) pg/ml
Major molecular form	Mature ANP	proBNP	NT-proBNP
Major sites of metabolism	Placenta and umbilical vessels	Not identified	Not identified
Mechanism of metabolism	NPR-C NEP digestion	NPR-C NEP digestion	Protease digestion
Stability	Unstable	Less stable	Stable
Use as a biomarker of fetal heart failure	Useful	Useful	Highly useful
Factors affecting concentrations	Fetal heart failure, tachyarrhythmias or bradyarrhythmias, right heart defects with moderate or severe TR, ventricular outflow tract obstruction without interventricular septum, preterm birth, and acidemia	Fetal heart failure, tachyarrhythmias or bradyarrhythmias, right heart defects with moderate or severe TR, ventricular outflow tract obstruction without interventricular septum, preterm birth, and acidemia	Fetal heart failure, tachyarrhythmias or bradyarrhythmias, right heart defects with moderate or severe TR, ventricular outflow tract obstruction without interventricular septum, preterm birth, and acidemia
**Amniotic fluid**			
Concentration, median (minimum–maximum)^#^	0.3 (0.2–9.8) pg/ml	3.9 (0.2–15.3) pg/ml	48 (7–1,329) pg/ml Approximately 1/30 of the umbilical vein level
Stability	Unstable	Unstable	Stable
Use as a biomarker of fetal heart failure	Not applicable	Not applicable	Extremely useful
Factors affecting concentrations	Not identified	Not identified	Fetal heart failure, tachyarrhythmias or bradyarrhythmias, right heart defects with moderate or severe TR, and gestational age

ANP, atrial natriuretic peptide; BNP, brain natriuretic peptide; CHD, congenital heart defect; NEP, neutral endopeptidase; NPR, natriuretic peptide receptor; NT-proBNP, N-terminal fragment of pro-brain natriuretic peptide; proANP: pro-atrial natriuretic peptide; proBNP; pro-brain natriuretic peptide; and TR, tricuspid valve regurgitation.

* Umbilical vein plasma (Miyoshi et al., 2018a).

**#Amniotic fluid (:** Miyoshi et al., 2018b).

Fetal tachyarrhythmia or bradyarrhythmia was strongly correlated with high NP levels (Miyoshi et al., 2018b). Abnormal venous Doppler sonography findings were more common and severe in fetuses with tachyarrhythmia or bradyarrhythmia than in those with CHD. Elevated NP levels are closely associated with abnormal venous Doppler findings, which indicate an increase in central venous pressure (Johnson et al., 1992). Elevated wall stress leads to cardiac remodeling and hypertrophy, which increases myocardial oxygen consumption and aggravates myocardial function. To overcome reduction in ventricular compliance, end-diastolic filling pressure, and hydrostatic central venous pressure increase to maintain cardiac output, thereby resulting in a higher release of NP from the fetal heart (Harada et al., 1998; Gardiner, 2005). Furthermore, our previous study found that NP levels in the umbilical cord blood reflect the severity of fetal arrhythmia and responses to fetal therapy. In the fetuses with tachyarrhythmias, NP levels in the responders of fetal therapy decreased to the levels similar to normal fetuses. Thus, NP concentrations can be used as biomarkers for the efficacy of fetal therapy (Miyoshi et al., 2019a). Similar to adults, damage to the ventricular wall in fetal tachyarrhythmia is reversible *in utero* (Gopinathannair et al., 2015). Meanwhile, the group with no indications for fetal therapy had significantly lower cardiovascular profile scores than the control group and had similar NP levels in the umbilical cord blood. Thus, NP levels complement echocardiographic assessment, and they may be useful in determining whether fetal treatment for arrhythmia is indicated.

Preterm birth and fetal acidemia are associated with high NP levels in fetuses with CHD and/or arrhythmia (Miyoshi et al., 2018b). Earlier studies have shown that gestational age is not an important determinant of ANP levels in fetuses and newborns (Kingdom et al., 1992; Ville et al., 1994). Plasma ANP levels in the umbilical cord blood were actually higher in fetuses with hydrops than in controls. Therefore, preterm birth caused by fetal heart failure or hydrops may contribute to high NP levels. A previous study has revealed that umbilical cord vein ANP levels were inversely correlated to umbilical artery pH (Kingdom et al., 1992). Maternal hypertensive disorder and fetal acidemia during labor stimulate fetal ANP production (Mäkikallio et al., 2001). In a recent research, high umbilical cord blood BNP levels and low pH might be associated with adverse outcomes in fetuses with CHD (Sahin-Uysal et al., 2020). Therefore, NP levels in the umbilical cord blood may be a useful surrogate marker of fetal maturation and antenatal stress (Kanbe et al., 2009).

### Amniotic Fluid NP Levels

In the amniotic fluid, NT-proBNP levels increase in a stepwise fashion with the severity of fetal heart failure in fetuses with CHD and/or arrhythmia (Miyoshi et al., 2018a). In contrast, ANP and BNP concentrations in the amniotic fluid are extremely low; hence, they are not good markers of fetal heart failure (Table 1). Although NT-proBNP is released from cardiomyocytes in equimolar amounts of BNP, it is not metabolized by the NP receptor C (NPR-C). Moreover, the half-life of NT-proBNP is significantly longer than that of BNP (McMurray et al., 2012). The glycosylation of NT-proBNP may further prevent metabolism *via* protease digestion in the amniotic fluid, similar in the blood (Hammerer-Lercher et al., 2008). Amniotic fluid NT-proBNP levels were strongly correlated with umbilical vein plasma NT-proBNP levels. Preterm birth, fetal tachyarrhythmias or bradyarrhythmias, and right heart defects with moderate or severe tricuspid valve regurgitation were associated with high amniotic fluid NT-proBNP levels, similar to the umbilical vein plasma NT-proBNP levels (Miyoshi et al., 2018b). Amniotic fluid NT-proBNP levels in fetuses with fetal tachyarrhythmias or bradyarrhythmias and right heart defects with moderate or severe tricuspid valve regurgitation were median 230 (range, 50–539) pg/ml and median 231 (range, 132–1,329) pg/ml, respectively, which were significantly higher than those of median 33 (range, 1–185) pg/ml in normal fetuses.

There are few data on the association between amniotic fluid NPs and fetal heart failure. Previous studies have reported a good correlation between amniotic fluid NT-proBNP levels and the severity of twin-twin transfusion syndrome in monochorionic diamniotic twin pregnancies (Bajoria et al., 2002; Delabaere et al., 2010; Habli et al., 2010; Van Mieghem et al., 2010). Both donor and recipient twins develop heart failure in severe twin-twin transfusion syndrome. However, BNP release is affected by factors such as fetal hypoxemia and renin transfer involving placental shunting from the donor to the recipient twin. Moreover, whether BNP production in the amniotic membrane is affected by polyhydramnios or oligohydramnios due to the twin-twin transfusion syndrome remains unclear. Our study focused on singletons with CHD and/or arrhythmia and systematically compared the association between amniotic fluid NP levels and fetal heart failure. We concluded that amniotic fluid NT-proBNP levels can reflect the severity of fetal heart failure (Miyoshi et al., 2018a).

Several studies have investigated NP levels in the umbilical cord blood and amniotic fluid upon delivery. The effects of maternal blood or vaginal secretion cannot be completely eliminated during amniotic fluid NP measurements, even though the mode of delivery and labor were not associated with NP concentrations (Miyoshi et al., 2018a). Percutaneous umbilical blood sampling or amniocentesis is required to provide real-time information and to identify therapeutic strategies with NPs in fetuses with CHD and/or arrhythmia. Ultrasonography is a non-invasive and repeatable investigation, while percutaneous umbilical blood sampling and amniocentesis are invasive and have several medical restrictions. However, amniocentesis is a common obstetric procedure that uses a hollow needle inserted into the uterus for screening chromosomal abnormalities in a fetus. Compared with percutaneous umbilical cord blood sampling, amniocentesis has a lower rate of complications and is technically easier to perform (Bigelow et al., 2016; Salomon et al., 2019). Our results will help to optimize the design of prospective studies using cardiovascular profile scores, and the measurement of NP concentrations in amniotic fluid samples collected *via* amniocentesis should be planned to identify the proper timing of delivery and improve the prognosis of fetuses with CHD and/or arrhythmia. Amniotic fluid NT-proBNP measurements are expected to complement the inadequate points of echocardiography in fetuses with CHD and/or arrhythmia.

## Production and Metabolism of NPs in the Fetoplacental Circulation

### Molecular Forms and Metabolism of NPs in the Fetoplacental Circulation

**Figure 1:**
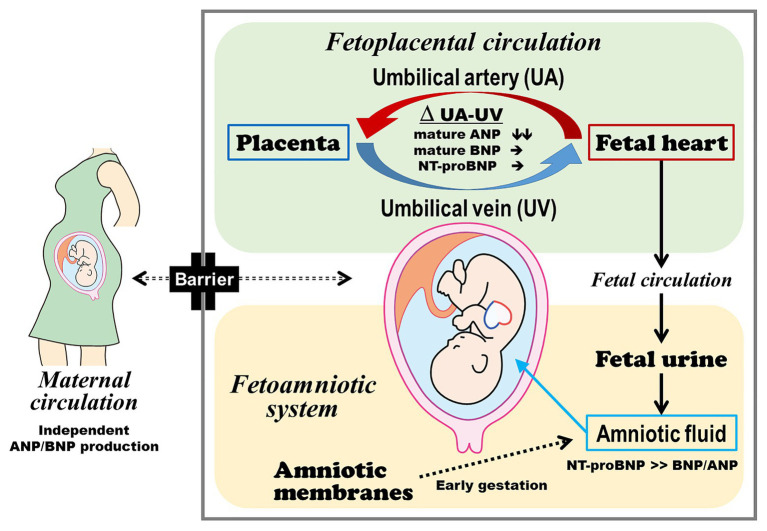
Production and metabolism of ANP, BNP, and NT-proBNP in the fetoplacental circulation and amniotic fluid. The fetus and mother independently produce and metabolize ANP and BNP. Fetal plasma ANP comprises the mature form, and the placenta and umbilical vessels can be the major sites of ANP metabolism. Fetal plasma BNP predominantly consists of the precursor form, which may reduce BNP metabolism in the fetoplacental circulation in addition to its lower affinity to natriuretic peptide receptor C. The plasma ANP, BNP, and NT-proBNP levels in the umbilical cord blood reflect the severity of heart failure. Amniotic fluid NT-proBNP derived from the fetal heart *via* fetal urine can be used to assess fetal heart failure at late preterm gestation or thereafter. ANP, atrial natriuretic peptide; BNP, brain natriuretic peptide; and NT-proBNP, N-terminal fragment of pro-brain natriuretic peptide.

A recent study has found the differential metabolism of ANP and BNP in the fetoplacental circulation (Miyoshi et al., 2019b; Figure 1). After passing through the placenta, the ANP levels in the umbilical vein plasma decreased to approximately one-half of the levels in the umbilical artery plasma in fetuses with CHD and/or arrhythmia and in controls. Thus, the placenta and umbilical vessels may be the major sites of ANP metabolism. Interestingly, previous reports showed that ANP, but not BNP, is expressed in the human placenta, particularly in cytotrophoblast cells (Lim and Gude, 1995; Graham et al., 1996). In our study cohort, there were several cases in which the ANP levels were higher in the umbilical vein than in the umbilical artery plasma. Hence, ANP may be secreted from the placenta locally or into the fetoplacental circulation (Miyoshi et al., 2019b) and may play a pivotal role in the regulation of fetoplacental hemodynamics.

By contrast, plasma BNP levels did not almost decrease after passing through the placenta, regardless of the type or presence of fetal heart disease. In adult cardiology, the half-life of BNP in the plasma is 10-fold longer than that of ANP (Mukoyama et al., 1991). Binding and internalization by the NPR-C and enzymatic degradation are the two main pathways involved in the clearance of circulating NPs (Nussenzveig et al., 1990; Norman et al., 1991). NPR-C-mediated degradation is the major mechanism responsible for the clearance of NPs from the circulation (Matsuo et al., 2019). The binding affinity of ANP to NPR-C is greater than that of BNP to NPR-C (Koller et al., 1991; Suga et al., 1992). Delayed BNP metabolism also reflects relative resistance to neutral endopeptidase, which is a major peptidase responsible for NP degradation (Smith et al., 2000; Walther et al., 2004). Therefore, the lower affinity of BNP to NPR-C and its resistance to neutral endopeptidase can make BNP more stable in the fetoplacental circulation.

Reverse-phase high-performance liquid chromatography revealed that in the fetoplacental circulation, ANP and BNP mainly comprised the mature and precursor forms, respectively (Miyoshi et al., 2019b; Table 1). In the adult circulation, ANP circulates as a mature alpha-ANP with full bioactivity, and BNP in the blood mainly comprises mature, fully active BNP-32 and weakly active its precursor proBNP (Matsuo et al., 2019). Recent studies have shown that glyco-proBNP – a glycosylated precursor – is a major circulating component, which results from impaired processing events by the glycosylation of threonine-71 of proBNP, in adults (Liang et al., 2007; Seferian et al., 2007; Semenov et al., 2009; Miller et al., 2011). ProBNP is highly glycosylated, and its properties are different from those of simple 108-residue proBNP peptide. The presence of highly glycosylated proBNP in the circulation may help to reduce BNP metabolism in the placenta and umbilical vessels since glycosylation generally provides protective effects against proteases (McCarthy et al., 2014). Differences in the circulating molecular forms are also responsible for the different properties in the metabolic clearance between ANP and BNP in the fetoplacental circulation. In adult patients with acute decompensated heart failure, self-compensation of myocardium for heart failure occurred by increasing mature BNP secretion *via* accelerating proBNP processing and activating the BNP/cGMP cascade (Takahama et al., 2018). Further studies should be conducted to validate the pathophysiology and prognostic value of different ANP and BNP molecular forms in fetuses with heart failure.

Several studies have shown that there is no or little exchange of ANP, BNP, and NT-proBNP across the placenta (Castro et al., 1989; Bar-Oz et al., 2005; Lechner et al., 2009). Fetus and mother secrete ANP and BNP independently of each other, and high NP levels in the umbilical cord plasma of fetuses with CHD and/or arrhythmia are primarily derived from the fetal heart (Miyoshi et al., 2019b). Therefore, the plasma concentrations of ANP and BNP in the fetoplacental circulation are likely to be regulated by the balance between production by the fetal heart and metabolism and clearance in the placenta and umbilical vessels.

### Origin of Amniotic Fluid NPs

The major source of NPs in the amniotic fluid has not yet been established (Figure 1). Fetal urine and lung fluid are contributors to amniotic fluid volume and NP concentrations (Merz et al., 2014; Carvajal et al., 2017). Amniotic membranes also produce and secrete NPs (Itoh et al., 1993; Carvajal et al., 2009). Gestational age should be considered in the assessment of amniotic fluid NT-proBNP concentrations. In normal fetuses with early gestation age, the amniotic membranes are the main source of NPs in the amniotic fluid. The reference values for amniotic fluid NT-proBNP in normal fetuses gradually decreased according to the progression of pregnancy, and it reaches a plateau after 34 weeks of gestation (Merz et al., 2014; Carvajal et al., 2017). A correlation between amniotic fluid and umbilical cord blood NT-proBNP concentrations was observed, even though the amniotic fluid had significantly lower NT-proBNP concentrations than the plasma (Miyoshi et al., 2018a). This correlation was similar to that between urinary and plasma NT-proBNP concentrations in adult patients with heart failure (Hammerer-Lercher et al., 2008). Taken together, at late preterm gestation or thereafter, amniotic fluid NT-proBNP is considered to be mainly derived from the fetal heart and can be used to assess fetal heart failure.

## Conclusion

Plasma NP levels in the umbilical cord blood reflect the severity of heart failure in fetuses with CHD and/or arrhythmia. Elevated NP levels are mainly attributed to an increase in central venous pressure secondary to arrhythmia or atrioventricular valve regurgitation caused by CHD. The plasma concentrations of ANP, BNP, and NT-proBNP in the umbilical cord blood had similar correlation profiles with the severity of fetal heart failure. Meanwhile, NT-proBNP levels in the amniotic fluid and umbilical cord blood are strongly correlated and amniotic fluid NT-proBNP levels increase according to the severity of fetal heart failure. In contrast, the ANP and BNP concentrations in the amniotic fluid are extremely low and, thus, are not good markers for assessing fetal heart failure.

The fetus and mother produce and metabolize NPs independently of each other. Metabolism in the fetoplacental circulation is quite different between ANP and BNP. Fetal plasma ANP comprises the mature form, and the placenta and umbilical vessels may be the major sites of ANP metabolism. Fetal plasma BNP predominantly consists of the precursor form, which may reduce BNP metabolism in the fetoplacental circulation in addition to its lower affinity to NPR-C.

The features of ANP, BNP, and their related peptides in the umbilical cord blood and amniotic fluid provided a strong basis for their use as biomarkers that can complement the inadequate points of ultrasonography.

## Author Contributions

TM drafted the manuscript. HH and NM edited and revised the manuscript. All authors contributed to the article and approved the submitted version.

### Conflict of Interest

The authors declare that the research was conducted in the absence of any commercial or financial relationships that could be construed as a potential conflict of interest.

## Abbreviations

NP: Natriuretic peptide
CHD: Congenital heart defect
ANP: Atrial natriuretic peptide
BNP: Brain natriuretic peptide
NT-proBNP: N-terminal fragment of pro-brain natriuretic peptide
NPR-C: NP receptor C
